# Need for cross-level iterative re-entry in models of visual processing

**DOI:** 10.3758/s13423-023-02396-x

**Published:** 2023-10-17

**Authors:** Thomas M. Spalek, K. P. Unnikrishnan, Vincent Di Lollo

**Affiliations:** 1 Department of Psychology, Simon Fraser University, 8888 University Drive, Burnaby, British Columbia V5A 1S6 Canada; 2 eNeuroLearn, Ann Arbor, MI USA

**Keywords:** Feed-forward processing, Re-entrant processing, Computational models, Biological models, Cross-level re-entry

## Abstract

Two main hypotheses regarding the directional flow of visual information processing in the brain have been proposed: feed-forward (bottom-up) and re-entrant (top-down). Early theories espoused feed-forward principles in which processing was said to advance from simple to increasingly complex attributes terminating at a higher area where conscious perceptions occur. That view is disconfirmed by advances in neuroanatomy and neurophysiology, which implicate re-entrant two-way signaling as the predominant form of communication between brain regions. With some notable exceptions, the notion of re-entrant processing has had a relatively modest effect on computational models of perception and cognition, which continue to be predominantly based on feed-forward or within-level re-entrant principles. In the present work we describe five sets of empirical findings that defy interpretation in terms of feed-forward or within-level re-entrant principles. We conclude by urging the adoption of psychophysical, biological, and computational models based on *cross-level* iterative re-entrant principles.

## Introduction

An issue that has generated considerable discussion in the fields of perception and cognition is the directional flow of information processing within the brain. Visual information processing has been modeled as a sequence of steps culminating in conscious awareness. Those models have been formulated in psychophysical, biological, and computational terms. Here, we examine the success of these models in accounting for the empirical evidence. Our principal objective was not to provide a comprehensive review of the literature. Rather, our approach was selective: studies were selected that were most pertinent to – and best illustrated – the specific issue under discussion.

## Abbreviated history of feed-forward and re-entrant models (psychophysics and biology)

Early psychophysical and biological theories of visual information processing expounded a feed-forward – also known as “bottom-up” – sequence in which the sensory input was said to advance from lower to higher processing levels culminating in a perception. A prime example is Selfridge’s (1959) Pandemonium model in which notional demons, each specializing in a different cognitive function, direct the incoming stimuli to progressively more complex higher-level demons converging to a decision-making demon that determines the observer’s conscious awareness.

In the 1960s and 1970s, feed-forward schemes such as Pandemonium were generally accepted as the default model of brain functioning. Their acceptance was supported by Hubel and Wiesel’s (1962, 1977) discovery of the feed-forward sequence of visual receptive fields aptly named Simple, Complex, and Hypercomplex. A benefit of feed-forward schemes lies in their simplicity and in their allowing subtraction procedures to calculate the timing of different processing stages (e.g., Donders, 1969).

Adequacy of the feed-forward scheme as a comprehensive theory was questioned by later advances in neuroanatomy and neurophysiology that revealed massive re-entrant pathways between brain regions (e.g., Felleman & Van Essen, 1991; Posner & Raichle, 1994; Zeki, 1993). If region A sends signals to region B, it is invariably the case that region B sends signals back to region A. Notably, the descending fibers are known to outnumber the ascending fibers and to be distributed widely, including into the spaces between the neurons at the lower level (e.g., Shipp & Zeki, 1989). Besides mediating a classical handshake with the units at the lower level, the widely distributed re-entrant signals can also bias the function of the lower-level units in preparation for the next step in the processing sequence (e.g., Sillito et al., 1994, see below). This anticipatory role of re-entrant processing has been incorporated into several models of information processing (e.g., Di Lollo et al., 2000; Hawkins & Blakeslee, 2004; Mumford, 1991, 1992).

Biological evidence notwithstanding, feed-forward principles continue to be implemented in theories of perception and cognition (e.g., de Waal & Ferrari, 2010).^1^ As noted in the next section, most *deep learning* computational models have also been based on exclusively feed-forward principles (e.g., Sejnowski, 2018).

## Abbreviated history of feed-forward and re-entrant models (computational)

The historical evolution of psychophysical/biological models is paralleled by the evolution of computational models. Early computational models employed strictly feed-forward architectures (McCulloch & Pitts, 1943; Hebb, 1949). Some of these models included the concept of *back propagation* (Rumelhart, Hinton, & Williams, 1985; Hecht-Nielsen, 1992) which may be regarded as involving re-entrant activity. We hasten to note, however, that *back propagation* cannot be regarded as the type or re-entrant activity that underlies perceptual and cognitive processes beyond the learning stage. This is because the re-entrant activity in back propagation mediates the *establishment* of a neural network with its hidden layers. Once established, however, that network functions in an exclusively feed-forward mode. After its establishment, a network may require updating by means of back propagation; once updated, however, that network continues to function in an exclusively feed-forward mode.

As a coda to the discussion on back propagation, we should note two ways in which the system may optimize the processing of the input. Back propagation can be regarded as a way of configuring the system in readiness for a given input. A similar objective is achieved in the laboratory by the instructions given to the observer. In both cases, incoming stimuli are processed “off-line” within a system whose configuration had been set before the arrival of the visual input. This way of configuring the system has been termed *task-set reconfiguration*.

In an alternative “on-line” procedure, the system’s configuration is altered as the input is being processed. An example of “on-line” processing has been proposed by Lamme and Roelfsema (2000; see below). In “on-line” processing the configuration of the system is not fixed as in “off-line” processing; rather, each step in the processing sequence is said to reconfigure the system in readiness for the next step. This sequence of automatic reconfigurations then leads to conscious awareness of the initial input. It needs to be emphasized that the present work deals exclusively with “on-line” processes.^2^

Returning to the discussion of models based on re-entry, it is important to distinguish re-entry *within* a given level in a multi-level system from re-entry *between* levels.^3^ Models based on within-level re-entry have been proposed by Fernandez et al. (Recurrent Multilayer Perceptron (RMLP), 1990), by Liang and Xiaolin (Recurrent Convolutional Neural Network (RCNN), 2015) and by Alom et al. (Inception Recurrent Convolutional Neural Network (IRCNN), 2021). The type of re-entry advocated in these models, however, is strictly *within* levels. This prevents them from accounting for the behavioural findings – discussed below – all of which involve re-entry *between* levels.

Models based on re-entry between levels have been proposed less frequently. An early instance was the fast-learning algorithm for deep belief nets (Hinton et al., 2006). That model contains multiple levels. The lower levels feed information forward to higher levels in an initial sweep but have no further feed-forward function. Rather, they convey only descending signals between levels. In contrast, the top levels exhibit full two-way connections between levels. Hinton et al.’s model was elaborated by Lee et al.’s (2011) Convolutional Deep Belief Network (CDBN) that postulated full two-way connections between all levels in the system. These between-level models are consistent with the empirical evidence discussed below.

In summary, there is no question that feed-forward processes are an essential part of perceptual and cognitive processes if for no other reason than to provide the initial sensory input to the system. Also, as discussed below in the context of face processing, they may underlie a distinct mode of information processing. But do they provide a suitable – or even acceptable – explanatory basis for the empirical findings? A negative answer to that question is demanded by a range of perceptual and cognitive phenomena that cannot be fully explained in terms of feed-forward processes or of processes constrained to re-entry *within* levels. Five such cases are reviewed below.

## Phenomena that require between-levels re-entrant accounts

### Metacontrast masking

Visual masking occurs when the perception of a target stimulus is impaired by the presentation of a subsequent visual stimulus (the mask). This form of masking is known as *backward* masking because the mask appears to act backwards in time. Two types of backward masking have been recognized, depending on the spatial relationship between the target and the mask: *pattern masking* and *metacontrast* masking. In pattern masking the contours of the mask are spatially superimposed on the target; in metacontrast masking the contours of the mask are closely adjacent to – but do not overlap – the contours of the target. Metacontrast is the main form of masking considered in the present work. For metacontrast masking to occur, the mask must follow the target in time. The optimal stimulus-onset asynchrony (SOA) between the target and the mask has been estimated to be about 100 ms in daylight viewing (Breitmeyer & Öğmen, 2006). Notably, no masking occurs when the target and the mask are displayed simultaneously.

Theoretical accounts of metacontrast masking have been formulated in terms of feed-forward processes. For example, a well-known theory proposed that the fast transient activity triggered by the onset of the mask overcomes and suppresses the slower sustained activity triggered by the target (Breitmeyer & Ganz, 1976). Here, we claim that feed-forward accounts of metacontrast masking are disconfirmed by recent evidence obtained in studies that used a variety of experimental paradigms. We begin with studies of event-related potentials (ERPs).

Fahrenfort, Scholte, and Lamme (2007a) recorded ERPs in a study of metacontrast masking. Observers were required to detect the presence of a square target figure that was followed (or not followed) by a metacontrast mask. The target was clearly visible when it was not masked but was invisible when followed by the mask. The corresponding ERPs revealed that the neural activity in the feed-forward sweep was the same when the target was masked as when it wasn’t masked. This indicates that no masking occurred in the feed-forward sweep. In contrast, the ERP components associated with the re-entrant activity – which were very much in evidence when the target was not masked – were entirely missing when the target was masked. This pattern of results strongly suggests that metacontrast masking acts by disrupting the re-entrant signals while leaving the feed-forward signals intact.

Further evidence that metacontrast masking depends critically on re-entrant processes has been reported by Fahrenfort, Scholte, and Lamme (2007b), who found that conscious awareness of the target in a metacontrast study correlated with re-entrant but not with feed-forward activity. More evidence along these lines has been reported by Lamme, Zipser, and Spekreijse (2002) and by Supèr, Spekreijse, and Lamme (2001), who found that feedback from extrastriate areas was critical for the stimuli to reach consciousness. Furthermore, Zhaoping and Liu (2022) found that metacontrast masking is weaker for stimuli displayed in the peripheral retina where feedback from higher to lower brain regions is thought to be weaker. Clearly, metacontrast masking cannot be wholly explained in terms of feed-forward processes.

### Object substitution masking

Object substitution masking (OSM) is also known as *common onset masking* because, unlike metacontrast masking, the target stimulus and the mask come into view simultaneously. The display consists of a target item, a variable number of distractor items, and a mask (typically, four small dots surrounding the target). No masking occurs if the entire display disappears after a brief exposure. Masking does occur, however, if the target and the distractors are removed after a brief exposure, and only the mask remains in view (Di Lollo, Enns, & Rensink, 2000; Lleras & Moore, 2003; Woodman & Luck, 2003).

On the strength of this evidence, Di Lollo et al. (2000) concluded that OSM cannot be explained by the kind of transient feed-forward activity that was held to account for metacontrast masking (Breitmeyer & Ganz, 1976; see above). This is because the simultaneous onset of the target and the mask in OSM precludes the mask from producing a separate onset transient that might suppress the ongoing sustained processing of the target. The conclusion that re-entry is involved in OSM has been corroborated by Boehler, Schoenfeld, Heinze, and Hopf (2008), who employed magnetoencephalography (MEG) to show that OSM is mediated by re-entrant activity to primary visual cortex.

### Figure-ground segregation

In a series of ingenuous experiments with awake monkeys, Lamme and Roelfsema (2000) investigated a train of visual processes that culminated in figure-ground segregation. They recorded the activity of neurons in primary visual cortex in response to a brief visual display. The display consisted of a square patch of oriented line segments on a background of line segments of the opposite orientation. The main finding was that re-entrant signals from extrastriate cortex altered the tuning of the neurons in V1 to perform several different functions in successive phases of the processing cycle. About 40 ms after stimulus onset the neurons were tuned to line orientation (loosely speaking, they acted as line-orientation detectors). About 40 ms later the same neurons became tuned to the subjective boundaries of the square patch (boundary detectors). Finally, about 40 ms after that, the same neurons became tuned to the square figure as distinct from the background (figure-ground detectors).

Ablation of extrastriate cortex caused the neurons to remain sharply selective for line orientation and figure boundaries, but the activity corresponding to figure-ground selection was missing. These findings confirm that, within a processing cycle, signals from higher centres re-enter the primary visual cortex and are essential in implementing the figure-ground selectivity of the neurons at the lower level. The re-entrant nature of the activity from extrastriate to striate cortex makes these results not amenable to accounts in terms of feed-forward processes.

### Enhancing the perception of directional motion

Another set of results that defies a feed-forward account has been reported by Sillito, Jones, Gerstein, and West (1994). The study involved monitoring the activity along the two-way

pathways between lateral geniculate nucleus (LGN) and primary visual cortex in the cat in response to moving gratings. The firing threshold of LGN neurons located just ahead in the motion path – but not yet activated by the moving grating – was significantly lowered by re-entrant signals from primary visual cortex.

Because of the lowered threshold, the primed neurons in LGN fired more readily and more strongly when eventually stimulated by the moving grating. As the authors note, this sequence of events may be regarded as the neurophysiological correlate of an expectation about the future location of a moving object. It goes without saying that this enhancement of motion processing in LGN stems exclusively from re-entrant signals between levels.

Homologous conclusions have been drawn from a series of experiments by Hupé et al. (1998), who studied the modulation of motion-selective units in Regions V1, V2, and V3 of macaque monkeys by re-entrant signals from Region V5. The main manipulation was to cool Region V5 to reduce the strength of re-entrant signals. The main finding was that the activity of neurons in the lower regions was reduced by as much as 95% when the activity of neurons in the higher region was suppressed by the reversible lesion. Clearly, feed-forward signals are not sufficient. Rather, appropriate functioning of motion-selective neurons in the lower regions depends critically on the re-entrant signals from higher areas. Beyond enhancing the functioning of neurons in the lower regions, Hupé et al. (1998) note that “… feedback projections serve to improve the visibility of features … in the stimulus and may thus contribute to figure–ground segregation, breaking of camouflage, and psychophysically demonstrated ‘pop-out’ effects” (p. 786).

### Face recognition

Feed-forward models encounter significant problems in modeling the findings in the face-recognition literature, especially those involved in identifying individual faces or specific facial expressions. We believe that those problems have arisen from the omission of re-entry as a critical factor in models of face recognition. Evidence consistent with the critical role of re-entry comes from recordings from temporal cortex of macaque monkeys (Sugase, Yamane, Ueno, & Kawano, 1999; Sugase-Miyamoto, Matsumoto, & Kawano, 2011). Specifically, Sugase et al. (1999) found that face recognition occurs in two distinct stages. In the words of Sugase et al. (1999, p. 869):

We found that single neurons conveyed two different scales of facial information in their firing patterns, starting at different latencies. Global information, categorizing stimuli as monkey faces, human faces or shapes, was conveyed in the earliest part of the responses. Fine information about identity or expression was conveyed later, beginning on average 51 ms after global information. We speculate that global information could be used as a ‘header’ to prepare destination areas for receiving more detailed information.

In agreement with Sugase et al. (1999), we suggest that generic faces are probably detected on the feed-forward sweep, perhaps along the dorsal pathway for the low spatial frequency contents of the image, as proposed by Bar et al. (2006). In contrast, identification of individual faces, or of specific facial expressions, requires re-entrant signalling from other cortical and subcortical brain regions. Sugase et al.’s findings should be considered in the broader context provided by Chow et al. (2022), in which different levels of categorization are shown to follow different time courses.^4^ Consistent with the theme of the present work, face perception cannot be wholly explained in terms of feed-forward processes alone.

## Concluding comments

Considerable evidence has been cited in the foregoing for phenomena that defy explanation in strictly feed-forward or within-level principles. Yet, despite this evidence, accounts of visual processing couched in feed-forward or within-level concepts continue to be proposed. For example, models based on essentially feed-forward principles can be found in a recent special issue of the journal *Vision Research* concerning deep neural network accounts of human vision (Heinke, Leonardis, & Leek, 2022).

On the other hand, the idea that between-levels re-entry is an important component of visual information processing has been around for some time. For example, Bridgeman (1980) anticipated the multiplexing function of re-entrant signals that was later proposed by Lamme and Roelfsema (2000, see above). In Bridgeman’s study monkeys performed a visual discrimination task under conditions of metacontrast masking. Consistent with Lamme and Roelfsema’s findings and conclusions, Bridgeman (1980, p. 347) proposed that “The results suggest an iterative or recurrent coding of visual information, where the same cells participate in early, late, and pre-response coding in different ways.”

Although most models of visual processing are couched in terms of feed-forward or within-level re-entrant processes, between-levels models offer a more realistic perspective. Among the latter class of models are the ALOPEX model of Harth, Unnikrishnan, and Pandya (1987), the ARTMAP model by Carpenter, Grossberg, and Reynolds (1991) and the CDBN model of Lee et al. (2009; described above). More recently, Hawkins and colleagues have put forth a systematic theory of brain functioning based on iterative re-entrant processes between levels (Hawkins & Blakeslee, 2004; Hawkins, Ahmad, & Cui, 2017; Hawkins, 2021).

In summary, models based on feed-forward or within-level re-entry principles cannot account for the empirical findings. In contrast, models based on iterative re-entry *between* levels offer a more promising perspective. To account for the empirical findings, however, such models need to include unique parameters tailor-made for each individual phenomenon. This said, the major objective of the present work was not to propose a novel model based on between-level re-entry. Rather, it was to draw attention to empirical findings that are beyond what can be explained in terms of feed-forward or within-level principles alone.

## Footnotes

1 Indeed, there has been at least some measure of reluctance in accepting the concept of re-entrant processing. At a major international conference, a speaker proffering a feed-forward model referred to the biological evidence for re-entry as an “evolutionary mistake.”

2 We thank Roberto Dell’Acqua for pointing out the distinction between “off-line” and “on-line” processing.

3 In biological settings, the term “level” refers to any given brain region. Whether a level is to be considered “high” or “low” depends on the context. Thus, a level defined as “low” in one context may be defined as “high” in a different context. This can best be illustrated by example. Pascual-Leone and Walsh (2001) used transcranial magnetic stimulation to study the timing and function of feedback between a relatively high-level region (V5) and a relatively low-level region (V1). Similarly, Sillito et al. (1994) investigated the temporal course of signals between a high-level region (V1) and a low-level region (Lateral Geniculate Nucleus). In computational settings, the term “level” has been used to refer to distinct processes performed at given stages of information processing. For example, McClelland and Rumelhart (1981, page 377) remarked as follows: …we assume that perceptual processing takes place within a system in which there are several levels of processing, each concerned with forming a representation of the input at a different level of abstraction. For visual word perception, we assume that there is a visual feature level, a letter level, and a word level, as well as higher levels of processing that provide "top-down" input to the word level. We thank an anonymous reviewer for requesting a more informative definition of the term “level” in both biological and computational contexts.

4 We thank an anonymous reviewer for noting the connection between the work of Sugase et al. (1999) and Chow et al. (2022).

## References

[CR1] Alom MZ, Hasan M, Yakopcic C, Taha TM, Asari VK (2021). Inception recurrent convolutional neural network for object recognition. Machine Vision and Applications.

[CR2] Bar M, Kassam KS, Ghuman AS, Boshyan J, Schmid AM, Dale AM, Hämäläinen MS, Marinkovic K, Schacter DL, Rosen BR, Halgren E (2006). Top-down facilitation of visual recognition. Proceedings of the National Academy of Sciences.

[CR3] Boehler CN, Schoenfeld MA, Heinze HJ, Hopf JM (2008). Rapid recurrent processing gates awareness in primary visual cortex. Proceedings of the National Academy of Sciences.

[CR4] Breitmeyer BG, Ganz L (1976). Implications of sustained and transient channels for theories of visual pattern masking, saccadic suppression, and information processing. Psychological Review.

[CR5] Breitmeyer B, Öğmen H (2006). *Visual masking: Time slices through conscious and unconscious vision*.

[CR6] Bridgeman B (1980). Temporal response characteristics of cells in monkey striate cortex measured with metacontrast masking and brightness discrimination. Brain Research.

[CR7] Carpenter GA, Grossberg S, Reynolds JH (1991). ARTMAP: Supervised real-time learning and classification of nonstationary data by a self-organizing neural network. Neural Networks.

[CR8] Chow JK, Palmeri TJ, Mack ML (2022). Revealing a competitive dynamic in rapid categorization with object substitution masking. Attention, Perception, & Psychophysics.

[CR9] de Waal FB, Ferrari PF (2010). Towards a bottom-up perspective on animal and human cognition. Trends in Cognitive Sciences.

[CR10] Di Lollo V, Enns JT, Rensink RA (2000). Competition for consciousness among visual events: The psychophysics of re-entrant visual processes. Journal of Experimental Psychology: General.

[CR11] Donders, F. C. (1969). Over de snelheid van psychische processen [On the speed of psychological processes]. (W. Koster, trans.). In W. Koster (Ed.), *Attention and performance: II*. Amsterdam: North-Holland (Original work published 1868).

[CR12] Fahrenfort JJ, Scholte HS, Lamme VA (2007). Masking disrupts re-entrant processing in human visual cortex. Journal of Cognitive Neuroscience.

[CR13] Fahrenfort JJ, Scholte HS, Lamme VA (2007). Perception correlates with feedback but not with feedforward activity in human visual cortex. Journal of Vision.

[CR14] Felleman DJ, Van Essen DC (1991). Distributed hierarchical processing in primate visual cortex. Cerebral Cortex.

[CR15] Fernandez, B., Parlos, A. G., & Tsai, W. (1990). *Nonlinear dynamic system identification using artificial neural networks (anns)* (pp. 133–141). In: International Joint Conference on Neural Networks.

[CR16] Harth E, Unnikrishnan KP, Pandya AS (1987). The inversion of sensory processing by feedback pathways: A model of visual cognitive functions. Science.

[CR17] Hawkins J (2021). *A thousand brains: A new theory of intelligence*.

[CR18] Hawkins, J., Ahmad, S., & Cui, Y. (2017). A theory of how columns in the neocortex enable learning the structure of the world. *Frontiers in neural circuits*, 81–98. 10.3389/fncir.2017.00081 PMC5661005 29118696

[CR19] Hawkins J, Blakeslee S (2004). *On intelligence*.

[CR20] Hebb DO (1949). The first stage of perception: Growth of the assembly. The Organization of Behavior.

[CR21] Hecht-Nielsen, R. (1992). Theory of the backpropagation neural network. In *neural networks for perception* (pp. 65-93). Academic Press.

[CR22] Heinke D, Leonardis A, Leek EC (2022). What do deep neural networks tell us about biological vision?. Vision Research.

[CR23] Hinton GE, Osindero S, Teh YW (2006). A fast learning algorithm for deep belief nets. Neural Computation.

[CR24] Hubel DH, Wiesel TN (1962). Receptive fields, binocular interaction and functional architecture in the cat’s visual cortex. Journal of Physiology, London.

[CR25] Hubel DH, Wiesel TN (1977). Functional architecture of macaque visual cortex. Proceedings of the Royal Society, London (B).

[CR26] Hupé JM, James AC, Payne BR, Lomber SG, Girard P, Bullier J (1998). Cortical feedback improves discrimination between figure and ground by V1, V2 and V3 neurons. Nature.

[CR27] Lamme VA, Roelfsema PR (2000). The distinct modes of vision offered by feedforward and recurrent processing. Trends in Neurosciences.

[CR28] Lamme VA, Zipser K, Spekreijse H (2002). Masking interrupts figure-ground signals in V1. Journal of Cognitive Neuroscience.

[CR29] Lee, H., Grosse, R., Ranganath, R., & Ng, A. Y. (2009, June). Convolutional deep belief networks for scalable unsupervised learning of hierarchical representations. In *Proceedings of the 26th annual international conference on machine learning* (pp. 609–616).

[CR30] Lee H, Grosse R, Ranganath R, Ng AY (2011). Unsupervised learning of hierarchical representations with convolutional deep belief networks. Communications of the ACM.

[CR31] Liang, M., & Xiaolin, H. (2015). *Recurrent convolutional neural network for object recognition*. Proceedings of the IEEE Conference on Computer Vision and Pattern Recognition: In.

[CR32] Lleras A, Moore CM (2003). When the target becomes the mask: Using apparent motion to isolate the object-level component of object substitution masking. Journal of Experimental Psychology: Human Perception and Performance.

[CR33] McClelland JL, Rumelhart DE (1981). An interactive activation model of context effects in letter perception: I. An account of basic findings. Psychological Review.

[CR34] McCulloch WS, Pitts W (1943). A logical calculus of the ideas immanent in nervous activity. The Bulletin of Mathematical Biophysics.

[CR35] Mumford D (1991). On the computational architecture of the neocortex I. The role of the thalamo-cortical loop. Biological Cybernetics.

[CR36] Mumford D (1992). On the computational architecture of the neocortex II. The role of cortico-cortical loops. Biological Cybernetics.

[CR37] Pascual-Leone A, Walsh V (2001). Fast backprojections from the motion to the primary visual area necessary for visual awareness. Science.

[CR38] Posner MI, Raichle ME (1994). *Images of mind*.

[CR39] Rumelhart, D. E., Hinton, G. E., & Williams, R. J. (1985). Learning internal representations by error propagation. In D. E. Rumelhart & i. L. McClelland (Eds.), *parallel distributed processing: Explorations in the microstructure of cognition. Vol. 1: Foundations*. Cambridge, MA: Bradford books/MIT press.

[CR40] Shipp S, Zeki S (1989). The organization of connections between areas V5 and V1 in the macaque monkey visual cortex. European Journal of Neuroscience.

[CR41] Sejnowski TJ (2018). *The deep learning revolution*.

[CR42] Selfridge O (1959). *Pandemonium: A paradigm for learning. In symposium on the mechanization of thought processes*.

[CR43] Sillito AM, Jones HE, Gerstein GL, West DC (1994). Feature-linked synchronization of thalamic relay cell firing induced by feedback from the visual cortex. Nature.

[CR44] Sugase Y, Yamane S, Ueno S, Kawano K (1999). Global and fine information coded by single neurons in the temporal visual cortex. Nature.

[CR45] Sugase-Miyamoto Y, Matsumoto N, Kawano K (2011). Role of temporal processing stages by inferior temporal neurons in facial recognition. Frontiers in Psychology.

[CR46] Supèr H, Spekreijse H, Lamme VA (2001). Two distinct modes of sensory processing observed in monkey primary visual cortex (V1). Nature Neuroscience.

[CR47] Woodman GF, Luck SJ (2003). Dissociations among attention, perception, and awareness during object-substitution masking. Psychological Science.

[CR48] Zeki S (1993). *A vision of the brain*.

[CR49] Zhaoping L, Liu Y (2022). The central-peripheral dichotomy and metacontrast masking. Perception.

